# Functional components of Chinese rice wine can ameliorate diabetic cardiomyopathy through the modulation of autophagy, apoptosis, gut microbiota, and metabolites

**DOI:** 10.3389/fcvm.2022.940663

**Published:** 2022-09-14

**Authors:** Jinjin Yang, Jiaoying Song, Jiedong Zhou, Hui Lin, Zhuonan Wu, Nan Liu, Wenqing Xie, Hangyuan Guo, Jufang Chi

**Affiliations:** ^1^Department of Cardiology, Shaoxing People's Hospital (Shaoxing Hospital, Zhejiang University School of Medicine), Shaoxing, China; ^2^Medical College of Shaoxing University, Shaoxing, China; ^3^Second Clinical Medical College of Zhejiang Chinese Medical University, Hangzhou, China

**Keywords:** diabetic cardiomyopathy, autophagy, apoptosis, gut microbiomes, metabolomics

## Abstract

**Background:**

Dietary polyphenols, polypeptides, and oligosaccharides modulate inflammation and immunity by altering the composition of gut microbiota. The polyphenols and polypeptides in Chinese rice wine have protective effects against cardiovascular disease. In this study, we hypothesized that the polyphenols, polypeptides, and oligosaccharides in Chinese rice wine can ameliorate diabetic cardiomyopathy (DCM) by altering gut microbiota and metabolites.

**Methods:**

Mice with DCM and high glucose cells were treated with rice wine polyphenols (RWPH), rice wine polypeptides (RWPE), and rice wine oligosaccharides. Cardiac function was evaluated by echocardiography and detection of myocardial injury markers. We observed the pathological structures using hematoxylin and eosin staining, Masson's trichrome staining, and transmission electron microscopy. The expression levels of autophagy-related proteins and stubRFP-sensGFP-LC3 fluorescence were measured to evaluate autophagy. We performed TUNEL staining and measured the levels of Bax, Bcl-2, and p53 to assess apoptosis. To analyze the effects of the rice wine functional components on the gut microbiota and metabolites of DCM mice, we performed fecal 16S-rDNA gene sequencing and serum untargeted metabolomics.

**Results:**

Our results showed an increase in cardiac and mitochondrial function, promotion of autophagy, and inhibition of cardiomyocyte apoptosis, which indicates that RWPH and RWPE can ameliorate DCM. The abundance of *Akkermansia* and *Desulfovibrio* were reduced by the presence of RWPH and RWPE. The growth of the *Lachnospiraceae*_NK4A136_group and *Clostridiales*-unclassified were promoted by the presence of RWPH. Tryptophan metabolism-associated metabolites were increased and phenylalanine levels were reduced by the presence of RWPH and RWPE. The biosynthesis of primary bile acids was enhanced by the presence of RWPH.

**Conclusion:**

Both RWPH and RWPE provided a protective effect against DCM by promoting autophagy, inhibiting apoptosis, and reversing both gut microbiota dysbiosis and metabolic dysregulation.

## Background

Diabetes is a very common health condition and a major global public health concern, and the prevalence of diabetes continues to rise worldwide. Diabetes affected approximately 537 million people in 2021, accounting for 10.5 % of the global population of adults (aged 20–79 years old). Approximately 6.7 million people died from diabetes and diabetic complications in 2021 (1), indicating a high rate of morbidity and mortality associated with the disease. The concept of diabetic cardiomyopathy (DCM) was first proposed by Ruler et al. in 1972, who described the postmortem findings of four patients with diabetic glomerulosclerosis who each had concomitant myocardial changes that could not be attributed to hypertension, coronary heart disease, excessive alcohol consumption, renal disease, or uremia (2). The minimum criteria for the diagnosis of DCM is left ventricular diastolic dysfunction and/or reduced left ventricular ejection fraction (LVEF), interstitial fibrosis, and pathological left ventricular hypertrophy (3). Although the exact pathogenesis of DCM is unclear, medical researchers suggest that key factors include oxidative stress, autophagy, apoptosis, formation of advanced glycation end products, and inflammation (4, 5).

Chinese rice wine is a traditional fermented drink with a history that spans more than 2,400 years in China. Chinese rice wine contains abundant functional components, including rice wine polyphenols (RWPH), rice wine polypeptides (RWPE), rice wine oligosaccharides (RWOL) and γ-aminobutyric acid (6). Research has demonstrated that RWPH and RWPE can inhibit homocysteine-induced proliferation and migration of vascular smooth muscle cells (7); additional studies have shown RWPH to have anti-hyperglycemic and anti-doxorubicin cardiotoxicity effects (8, 9). Dietary polyphenols, polypeptides, and oligosaccharides modulate inflammation and immunity by altering the composition of gut microbiota (10–13).

The human gut is host to 100 trillion microorganisms across more than 1,000 different microbial species (14, 15). A symbiotic relationship exists between the intestines of healthy individuals and their gut microbiota and has an important role in the health of metabolism, immunity, and intestinal protection (16). Cardiovascular diseases, such as heart failure, hypertension, and atherosclerosis, can alter the composition of gut microbiota; recent research has focused on the ways that gut microbiota may contribute to the development of cardiovascular disease (17, 18).

In this study, we aim to determine whether the functional components of Chinese rice wine provide cardioprotective effects against DCM. We further aim to establish if the functional components of Chinese rice wine can affect the relationship between DCM and gut microbiota and metabolites.

## Methods

### Animals and treatments

Twenty four male db/db and 6 db/m mice (7 weeks old) were obtained from the Model Animal Research Center of Nanjing University (Nanjing, China). All animal protocols were approved by the Animal Care and Use Committee of Shaoxing Hospital, Zhejiang University School of Medicine, according to the Guide for the Care and Use of Laboratory Animals of the National Institutes of Health. Animals were kept in the Animal Center of Shaoxing Hospital, Zhejiang University School of Medicine at room temperature (20°C), relative humidity of 50–60 %, and subjected to a 12 hour light/dark cycle. After acclimatization for 1 week, db/m mice were treated as the control (CON) group (*n* = 6). We randomly divided the db/db mice into four groups (*n* = 6 per group): the DCM group, the DCM + RWPH group, the DCM + RWPE group, and the DCM + RWOL group. Throughout the experiment, the CON group was fed normal chow, and the other four groups were fed a high-fat diet (HFD, Research Diet D12492). After 4 weeks, the mice with a fasting blood glucose ≥ 11.1 mmol/L were considered as successful diabetic mouse models (19). The diabetic mice groups were respectively treated with RWPH (gavage at 1.5 mg/kg), RWPE (gavage at 0.5 g/kg), RWOL (gavage at 0.3 mg/kg), or vehicle (saline, gavage) once daily (20) for 18 weeks. We used echocardiography to determine the cardiac function of all mice. After mice were sacrificed, we collected samples of feces, blood, and myocardial tissue for subsequent experiments.

### Reagents

The RWOL used in this experiment consisted of panose, isomaltose, and isomaltotriose, with a mass ratio of 3.14:3.96:0.14 (7). Panose, isomaltose, and isomaltotriose were purchased from the Macklin Biochemical Company (Shanghai, China). RWPH and RWPE extracted from Chinese rice wine were provided by the Academy of Chinese Medical Science at Zhejiang Chinese Medical University (Hangzhou, China).

### Transthoracic echocardiography

Mice were anesthetized with isoflurane, then we monitored the left ventricular fractional shortening (LVFS), LVEF, left ventricular end-diastolic volume, and left ventricular end-systolic volume using a Philips iE33 system with an s5-1 probe (12-14 MHz) (Philips Medical, Best, Netherlands).

### Transmission electron microscopy

The myocardial tissue at the apex of the heart was cut into 1.0 mm fragments. The fragments were fixed in 2.5 % glutaraldehyde at 4°C for 2 h. After rinsing with phosphate buffer, the samples were fixed in 1 % osmic acid for 1 h followed by dehydration using a series of acetone washes. Tissues were embedded, cut into ultrathin sections, and stained with toluidine blue. The slides were observed under Cs-corrected transmission electron microscope with a monochromator (Titan G2 60-300, FEI).

### Histopathological examination

Tissue specimens were fixed with 10 % formalin, embedded in paraffin, and then cut into 5-μm-thick sections. Sections were stained with hematoxylin and eosin and Masson's trichrome, then imaged using a Leica DM 3000 biological microscope (Leica, Wetzlar, Germany) to evaluate the myocardial morphology and collagen deposition.

### Serum biochemical analysis

We drew blood samples from the inferior vena cava, then centrifuged the samples at 3,000 rpm for 10 min to obtain the serum. The serum levels of lactate dehydrogenase, creatine kinase-MB, and low-density lipoprotein cholesterol were measured using corresponding detection kits (Nanjing Jiancheng Bioengineering Institute, Nanjing, China).

### Cell culture and treatments

We purchased H9C2 cells from the Chinese Academy of Sciences Cell Bank (Shanghai, China). We cultured the H9C2 cells in DMEM (Sigma, Shanghai, China) with 10 % FBS (Gibco, Shanghai, China) and 1 % streptomycin-penicillin (Beyotime, Jiangsu, China) at 37 °C in a 5 % CO_2_ atmosphere. The cells were incubated with a high glucose (HG) concentration (30 mM) to establish a glucotoxicity model. To investigate the role of Chinese rice wine functional components, the cells were divided into five groups that were cultured with different treatments: CON (5.5 mmol/L glucose medium), HG (30 mmol/L glucose medium), HG + RWPH (30 mmol/L glucose medium + 50 mg/L RWPH), HG + RWPE (30 mmol/L glucose medium + 20 g/L RWPE), and HG + RWOL (30 mmol/L glucose medium + 10 g/L RWOL). The doses of rice wine derived functional components were based on the results of our previous experiments (7). In another part of the experiment, in order to explore the relationship between apoptosis and autophagy, the cells were divided into six groups: the CON, HG, HG + RWPH, HG + RWPE, HG + RWPH + 3-Methyladenine(3-MA, an autophagy inhibitor, MedChenExpress, Shanghai, China) and HG + RWPE + 3-MA group. 3-MA was dissolved to 5 mM for treatment. The intervention for the rice wine functional components and 3- MA were both 2 days. Cell experiments of each group were repeated in three times.

### Western blot analysis

Protein samples were extracted from myocardial tissue and H9C2 cells. Protein concentrations were determined using the BCA method to ensure consistent loading of each sample. Proteins were separated using SDS-PAGE, and then transferred to 0.45-μm PVDF membranes. After blocking with skim milk for 1 hour, membranes were incubated with antibodies against Bcl-2 (ab32124), Bax (ab53154), p53 (ab32389), LC3B(ab63817), SQSTM1/62 (ab91526), ATG5 (ab53154), Beclin1 (ab62557), or β-actin (ab8226) (antibodies purchased from Abcam Biotechnology, USA) at 4 °C overnight. After labeling with secondary antibodies, the target protein was observed using enhanced chemical luminescence.

### Stably expressing stubRFP-sensGFP-LC3

We purchased tandem stubRFP-sensGFP-LC3 lentiviruses (Genechem Company, Shanghai, China) to monitor autophagy flux. Well-conditioned cells were seeded into 6-well plates and transfected with stubRFP-sensGFP-LC3 lentiviruses for 24 hours. Cells stably expressing stubRFP-sensGFP-LC3 were selected using puromycin (5 ug/ml) treatment. After the corresponding intervention for 2 days, cells were observed using a Nikon Eclipse Ti-U fluorescence microscope.

### TUNEL staining

We conducted TUNEL assays using an *in-situ* cell death detection kit (Roche Co. Ltd., Basel, Switzerland), which detects DNA fragmentation in individual cells. Paraffin-embedded cardiac tissue sections were dewaxed with xylene and rehydrated using a graded ethanol series. Sections were incubated with proteinase K for 20 min at 37 °C. Sections were then incubated with the TUNEL reaction mixture according to the manufacturer's instructions. After incubation with DAPI for 3 min, the number of TUNEL-positive cells were counted under a Nikon Eclipse Ti-U fluorescence microscope at 200 × magnification.

### Statistical analysis

SPSS 23.0 (SPSS Inc., Chicago, IL) was used for statistical analyses. All data are presented as the mean ± SEM. Multiple groups were analyzed using a one-way ANOVA. ROC analysis was used to identify the metabolites that contributed the most to the differences between groups. Correlations between the selected metabolites and bacterial taxa were assessed using the Spearman correction analysis. Charts were created using Prism 8 (San Diego, CA, USA). Non-normally distributed data were analyzed using the Wilcoxon rank-sum test and Kruskal-Wallis test. Statistical significance was set at P < 0.05.

## Results

### RWPH and RWPE ameliorate cardiac function in DCM mice

**Figure 1:**
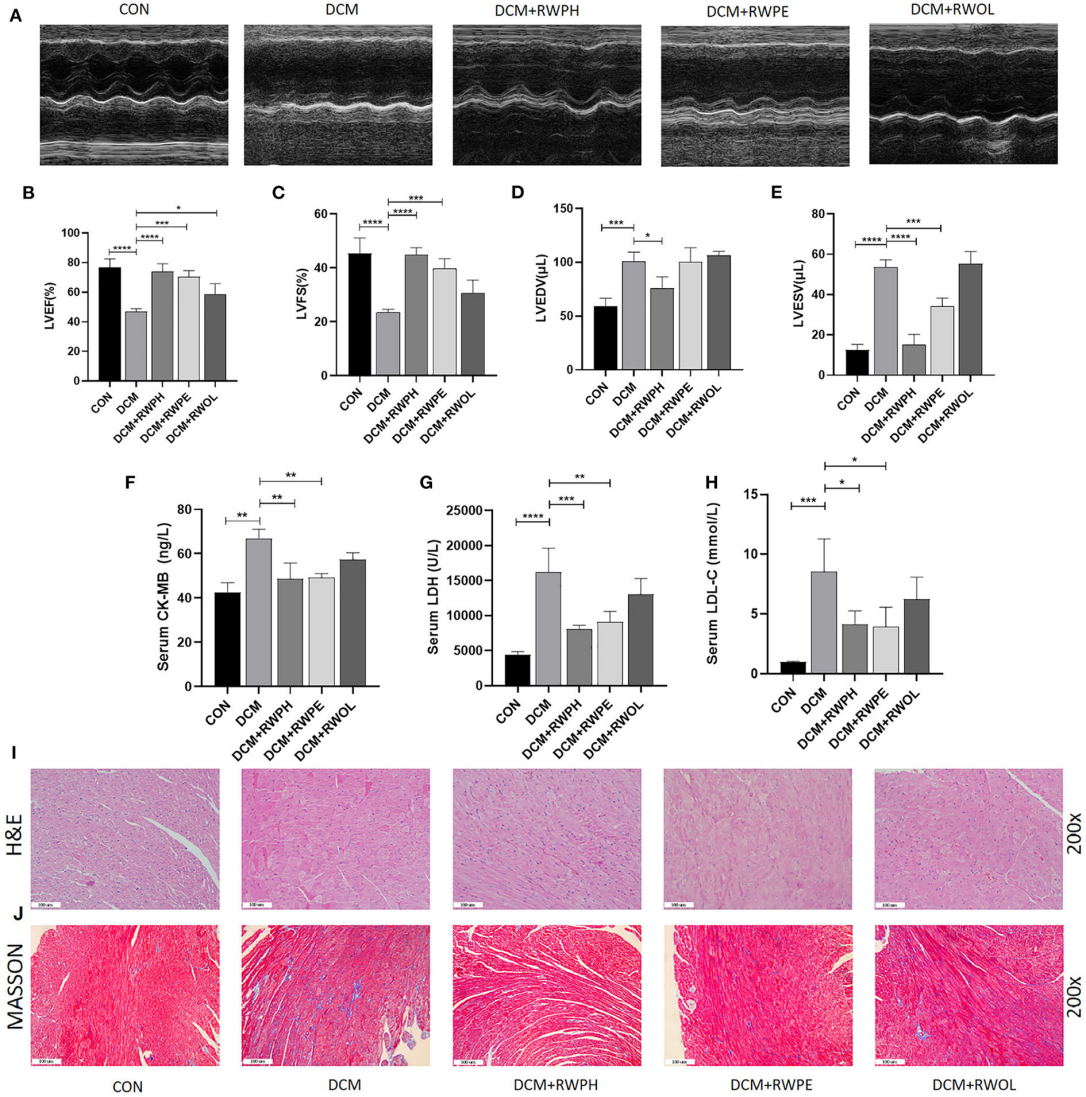
RWPH and RWPE ameliorate cardiac function and alleviate cardiac fibrosis and cardiomyocyte hypertrophy in DCM mice. **(A)** M-mode echocardiography and corresponding images of each group mice hearts. **(B–E)** Results of **(B)** left ventricular ejection fraction (LVEF), **(C)** left ventricular fractional shortening (LVFS), **(D)** left ventricular end-diastolic volume (LVEDV) and **(E)** left ventricular end-systolic volume (LVESV) in each group mice. **(F–H)**, Results of **(F)** serum creatine kinase-MB (CK-MB), **(G)** serum lactate dehydrogenase (LDH) and **(H)** serum low-density lipoprotein cholesterol (LDL-C) in each group mice. **(I)** Hematoxylin and eosin (H&E) staining of the left ventricular tissue section in each group mice. **(J)** Masson trichrome stain of the left ventricular tissue section in each group mice. ^*^P < 0.05, ^**^P < 0.01, ^***^P < 0.001, and ^****^P < 0.0001.

We performed echocardiography to assess the efficacy of rice wine functional components after 18 weeks of mice undergoing treatment. Using the CON group as the base comparison, impaired cardiac function was observed in the DCM group with decreased levels of LVFS and LVEF and increased levels of left ventricular end-diastolic volume and left ventricular end-systolic volume. These negative changes were reversed after the mice were received treatment with RWPH and RWPE (Figures 1A–E); however, treatment with RWOL had a negligible effect on the restoration of cardiac function. Additionally, we detected myocardial injury markers and low-density lipoprotein in the serum (Figures 1F–H). Lactate dehydrogenase, creatine kinase-MB, and low-density lipoprotein levels in DCM mice were significantly higher than those in CON mice. Similarly, these negative indicators were significantly decreased after treatment with RWPH and RWPE, whereas treatment with RWOL had no effect.

### RWPE and RWPH alleviate cardiac fibrosis and cardiomyocyte hypertrophy in DCM mice

Using hematoxylin and eosin staining, we observed the occurrence of cardiac hypertrophy and obscure intercellular borders in the diabetic myocardial tissue, which represented the onset of myocardial necrosis (Figure 1I). Irregular and increased myocardial fibers of myocardial tissue in the DCM group were observed by Masson staining (Figure 1J). These results clearly show that treatment with RWPE and RWPH significantly reversed pathological changes.

### RWPH and RWPE enhance autophagy in DCM mice

**Figure 2:**
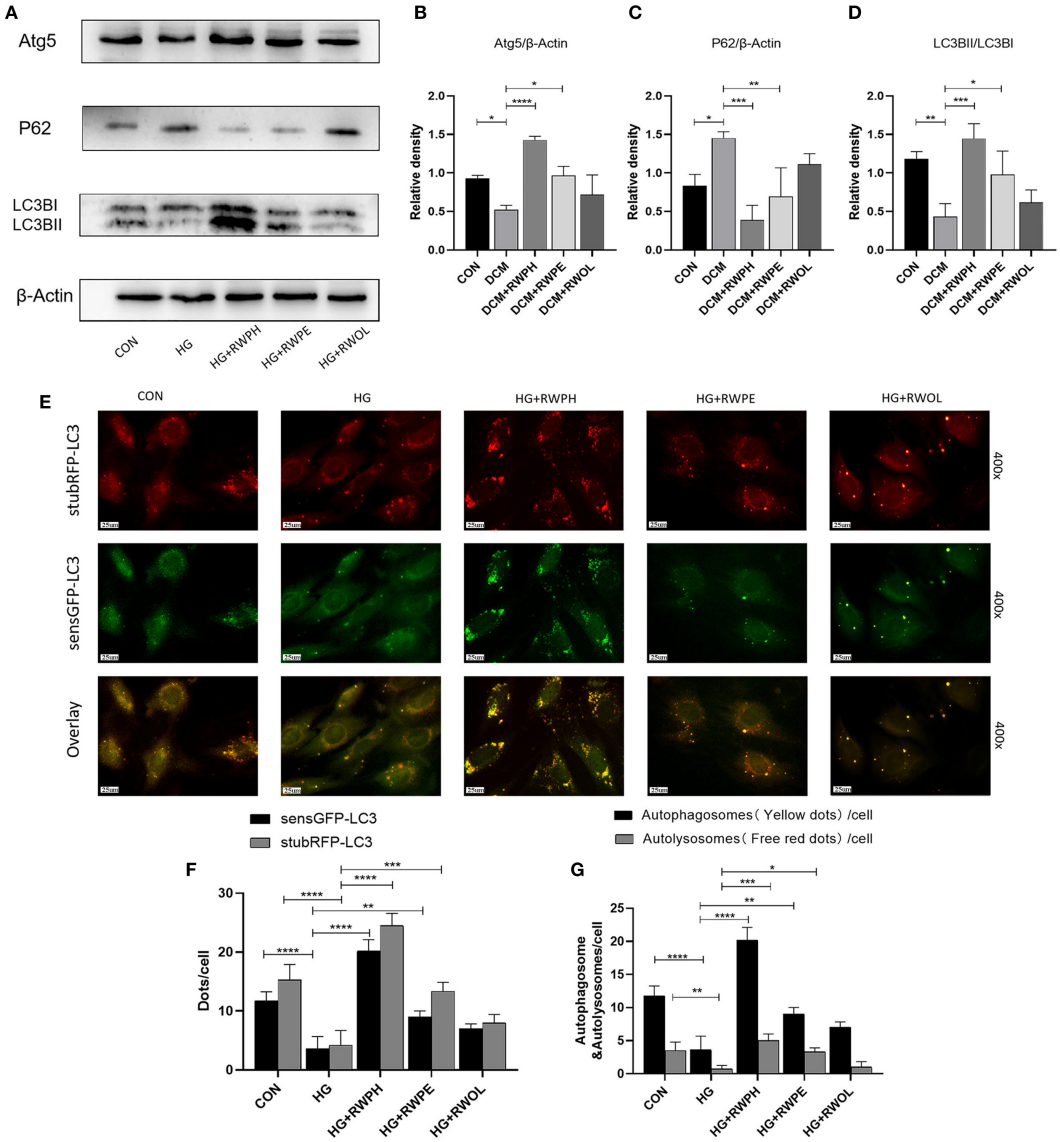
RWPH and RWPE rescue impaired autophagy in high-glucose cultured H9C2 cells. **(A–D)** Western blot analysis of the relative protein expression of ATG5, P62, and LC3B in H9C2 cells. **(E–G)** stubRFP-sensGFP-LC3 fluorescence was used to determine the autophagic activity in HG group and treatment groups. GFP fluorescence represents autophagosomes, RFP fluorescence represents autophagosomes and autolysosomes, the two merged to form yellow dots represent autophagosomes, and the remaining red dots represent autolysosomes. *P < 0.05, **P < 0.01, ***P < 0.001, and ****P < 0.0001.

H9C2 cells that were cultured with 30 mmol/L glucose exhibited autophagy defects (decreased LC3BII/LC3BI ratio, low ATG5 expression, and high SQSTM1/p62 accumulation) (Figures 2A–D). In comparison to that of the HG group, treatment with RWPH and RWPE increased the LC3BII/LC3BI ratio and the ATG5 expression and decreased the SQSTM1/p62 expression. For further validation, we observed the dynamic changes in autophagy by transfecting stubRFP-sensGFP-LC3 lentiviruses to assist with the differentiation between autophagosomes and autolysosomes (21). The green dots shown by GFP fluorescence represent autophagosomes, and the red dots exhibited by RFP fluorescence represent autophagosomes and autolysosomes. In the merged picture, the yellow dots represent autophagosomes and the individual red dots represent autolysosomes. In Figures 2E–G, the red and yellow dots in the HG group rapidly decreased, whereas sustained treatment with RWPH and RWPE resulted in a notable increase in the number of these dots.

**Figure 3:**
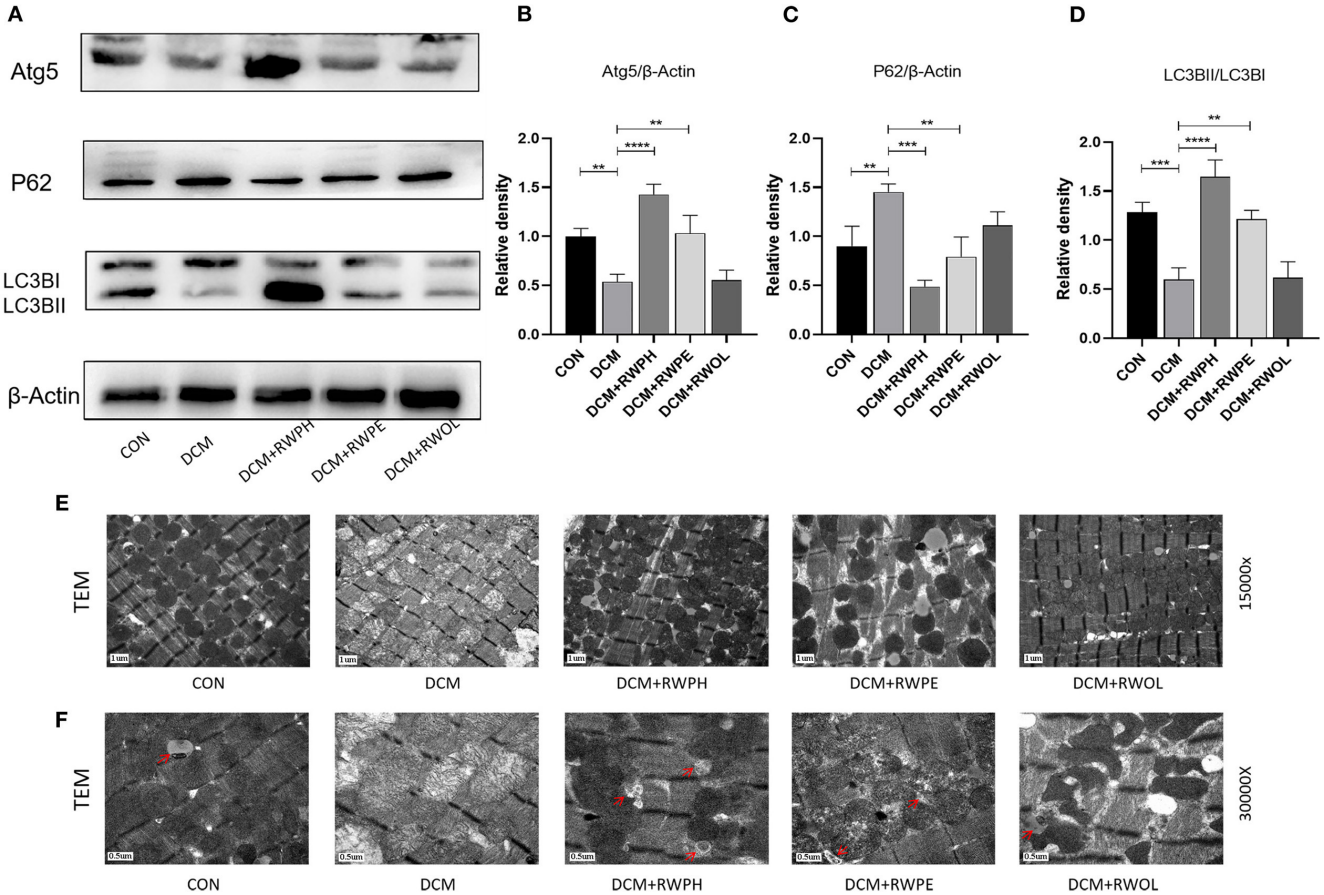
RWPH and RWPE clear dysfunctional mitochondria through enhanced autophagy in DCM mice. **(A–D)** Western blot analysis of the relative protein expression of ATG5, P62, and LC3B in myocardial tissue. **(E)** Transmission electron microscopy of left ventricular specimens showing mitochondria with different pathological forms. **(F)** the autophagosomes of each group under transmission electron microscopy, which are pointed out by red arrows. **P < 0.01, ***P < 0.001, and ****P < 0.0001.

To explore changes in autophagy *in vivo*, we extracted proteins from the hearts of mice. Figures 3A–D shows results that support those of the *in vitro* experiments: a decreased LC3B II/LC3B I ratio and ATG5 expression and increased SQSTM1/p62 levels in the DCM group; the 18-week intervention with RWPH and RWPE reversed autophagy defects in the DCM group. Impaired autophagy leads to mitochondrial dysfunction and accumulation of damaged mitochondria (22). Using transmission electron microscopy, we observed mitochondrial swelling, vacuolar degeneration, and cristae lesions in the DCM group; RWPH and RWPE treatment ameliorated these pathological changes (Figure 3E). In addition, autophagosomes were increased after treatment of RWPH and RWPE compared with DCM group (Figure 3F). Our results suggest that dysfunctional mitochondria were cleared and replaced by nascent mitochondria after treatment.

### RWPH and RWPE relieve cardiac apoptosis in DCM mice

**Figure 4:**
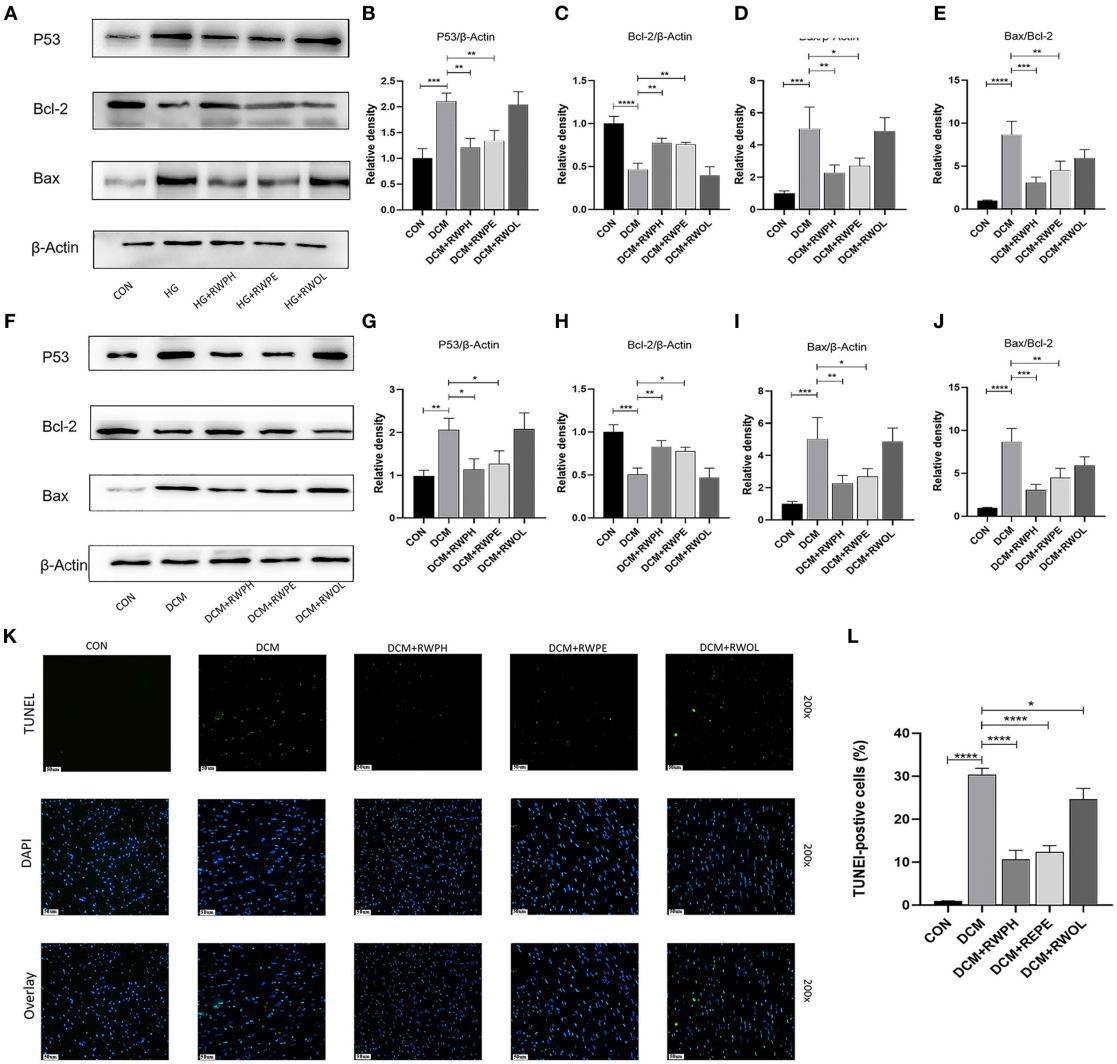
RWPH and RWPE relieved apoptosis in DCM mice and high-glucose cultured H9C2 cells. **(A–E)** Western blot analysis of the relative protein expression of P53, Bcl-2, and Bax in H9C2 cells. **(F–J)** Western blot analysis of the relative protein expression of P53, Bcl-2, and Bax in myocardial tissue. **(K,L)** TUNEL assay was used to determine the apoptotic rate in myocardial tissue of different groups. *P < 0.05, **P < 0.01, ***P < 0.001, and ****P < 0.0001.

Increased cardiac apoptosis is considered a major risk factor for the development of DCM (23); we, thus, explored the effects of rice wine functional components on cell apoptosis in DCM mice. In the *in vitro* experiments, the Bax/Bcl-2 ratio and levels of apoptosis-related proteins, such as Bax and p53, were increased in the HG group but were decreased in both the HG + RWPH group and the HG + RWPE group (Figures 4A–D). HG stimulation decreased the expression level of anti-apoptotic genes (such as Bcl-2), whereas treatment with RWPH and RWPE reversed these changes significantly. The protein expression level in the HG + RWOL group was similar to that in the HG group.

Consistent with the results of the *in vitro* experiments, treatment with RWPH and RWPE significantly alleviated the increase in Bax/Bcl-2 ratio, Bax and p53 as well as the decrease in Bcl-2 in DCM mice (Figures 4E–H). To seek further verification, we used TUNEL staining to detect if myocardial apoptosis was occurring in myocardial tissue. Confirming the *in vitro* results, we found that the number of green-fluorescent apoptotic cells in the DCM group was significantly higher than that in the CON group, and treatment with RWPH and RWPE markedly attenuated the apoptosis (Figures 4I,J).

### Anti-apoptotic effects of RWPH and RWPE can be inhibited by autophagy inhibitor

**Figure 5:**
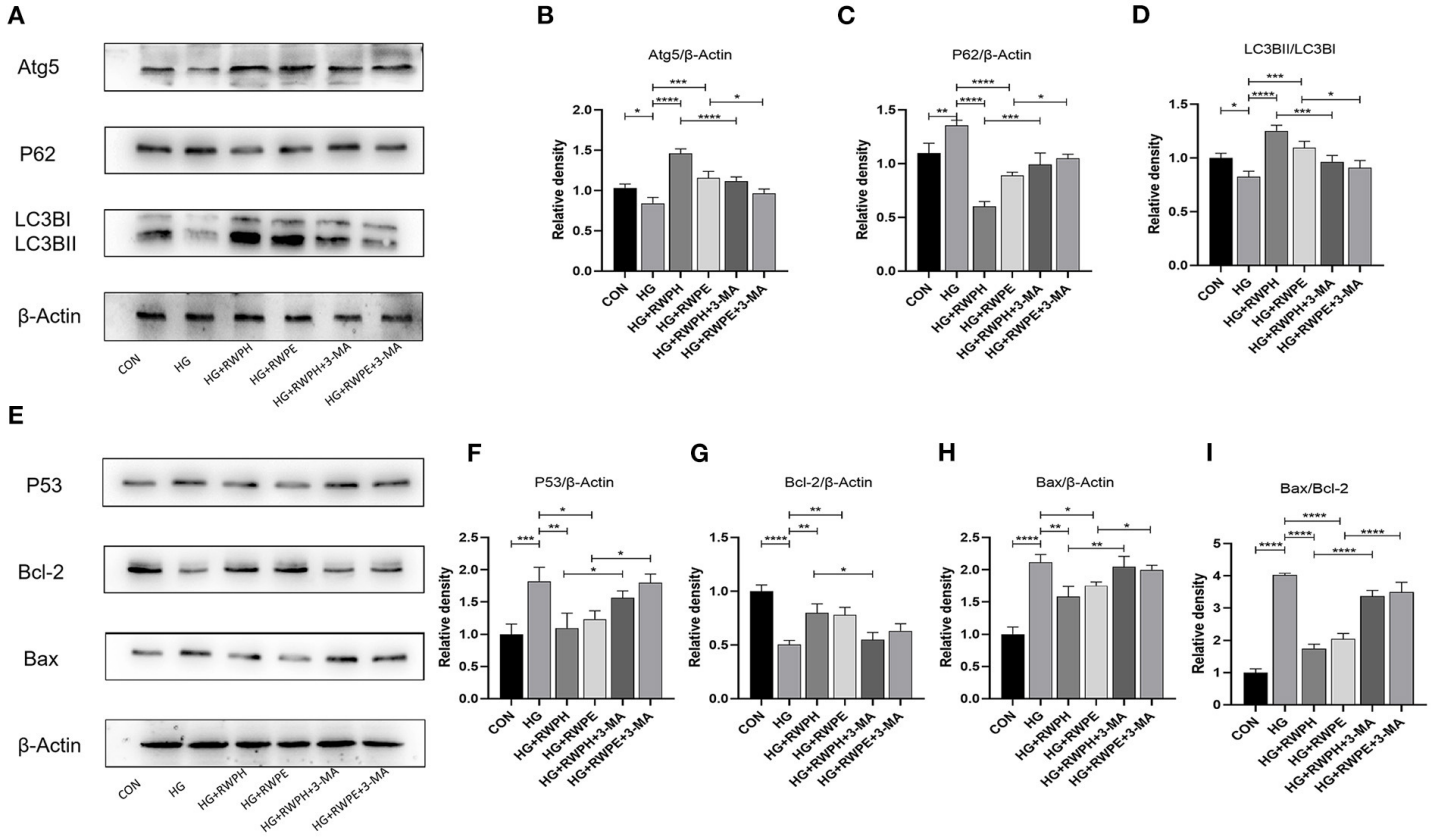
Autophagy inhibitor was able to counteract the anti-apoptotic effects of RWPH and RWPE. **(A–D)** Western blot analysis of the relative protein expression of ATG5, P62, and LC3B in each group. **(E–I)** Western blot analysis of the relative protein expression of Bcl-2, P53 and Bax in each group. *P < 0.05, **P < 0.01, ***P < 0.001, and ****P < 0.0001.

To evaluate whether the anti-apoptotic effects of RWPH and RWPE are related to their promotion of autophagy, we introduced 3-MA, an autophagy inhibitor, and set HG+RWPH+3-MA group and HG+RWPE+3-MA group. As expected, 3-MA as an autophagy inhibitor could effectively inhibit the autophagy-promoting effect of RWPH and RWPE. Compared with the HG+RWPH and HG+RWPE groups, the LC3BII/LC3BI ratio and ATG5 expression in the 3-MA intervention group decreased, whereas SQSTM1/p62 expression increased (Figures 5A–D).

Then, we detected apoptosis-related proteins. According to Figures 5E–I, the decreased autophagy was able to counteract the anti-apoptotic effects of RWPH and RWPE, evidenced by the higher ratio of Bax/Bcl-2 and level of P53 expression in HG+RWPH+3-MA group and HG+RWPE+3-MA group compared with the RWPH or RWPE alone.

### RWPH and RWPE alter the composition of gut microbiota in DCM mice

We performed 16S-rDNA gene sequencing on the stool samples of mice in each group (*n* = 4) to explore the role of gut microbiota in mediating HG-induced cardiotoxicity and how the functional components of rice wine may be regulating the composition of gut microbiota. The alpha diversities of the analyzed gut microbiota were the Chao1 (microbial community richness) and Shannon (microbial community diversity) indices.

**Figure 6:**
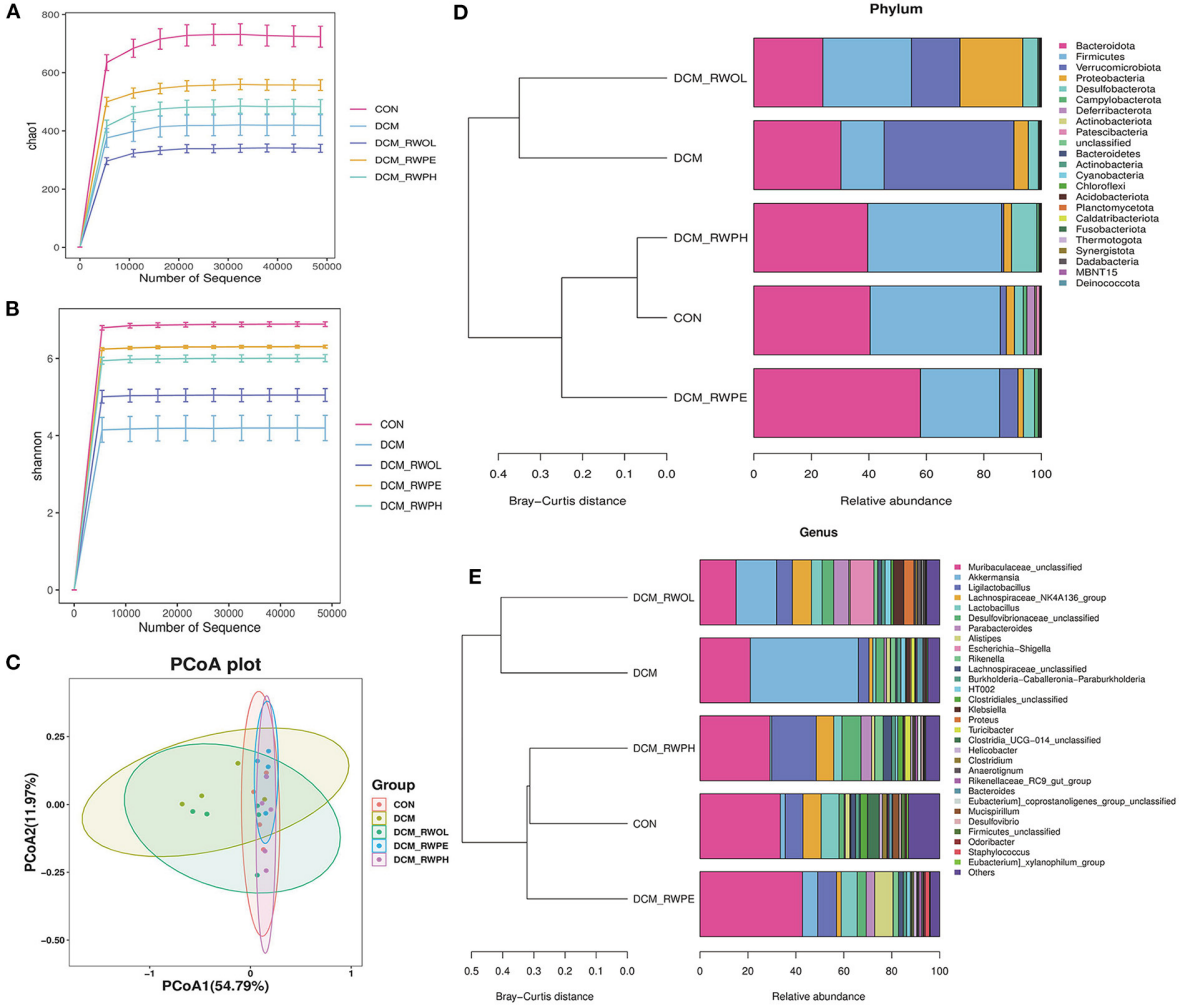
RWPH and RWPE prevented gut microbiota dysbiosis in DCM mice (n=4 per group). **(A,B)** The Chao1 and Shannon diversity index of intestinal bacteria was examined by 16S-rRNA high-throughput sequencing. **(C)** The β-diversity of intestinal bacteria was compared by the weighted unifrac principal coordinates analysis (PCoA). **(D)** At the phylum level, the Bray-Curtis distance clustering tree and the relative abundance of the gut bacterial in each group. **(E)** At the genus level, the Bray-Curits distance clustering tree and the relative abundance of the gut bacterial in each group.

The results of the Chao1 index showed that the gut microbiota community richness of the DCM group was lower than that in the CON group (Figure 6A). Similarly, the DCM + RWPH group and the DCM + RWPE group both showed a higher community richness than that of the DCM group. The richness of the DCM + RWOL group was the lowest.

The results of the Shannon index showed that the gut microbiota community diversity was lower in the DCM group than in the CON group; the decrease was attenuated by treatment with rice wine functional components (Figure 6B). Beta diversity (using principal coordinate analysis) refers to species differences between different environmental communities. Weighted UniFrac principal coordinate analysis revealed significant clustering of microbiota composition in the CON group, the DCM + RWPH group, and the DCM + RWPE groups. Contrastingly, both the DCM group and the DCM + RWOL group showed a marked difference (Figure 6C). The left part of Figure 6D shows the Bray-Curtis distance clustering tree (the shorter the branch, the more similar the gut microbiota composition). At the phylum level, the composition of gut microbiota in both the DCM group and the DCM + RWOL group were similar. The other groups were close, especially the CON group and the DCM + RWPH group. The right part of Figure 6D shows the relative abundance of gut microbiota for each group, the DCM group measured a lower abundance of *Bacteroidetes* and *Firmicutes* and higher abundance of *Proteobacteria* and *Verrucomicrobiota* compared to that of the CON group.

The alteration in the abundance of gut microbiota at the phylum level in DCM mice was alleviated by RWPH and RWPE to varying degrees. As shown in Figure 6E, at the genus level, the Bray-Curtis distance clustering tree showed the same results as in Figure 6D. Compared to that of the CON group, the abundance of *Muribaculaceae*-unclassified, *Ligilactobacillus*, and *Lachnospiraceae*_NK4A136_group decreased whereas that of *Akkermansia* increased in the DCM group. The treatment of rice wine functional components partially reversed these changes.

**Figure 7:**
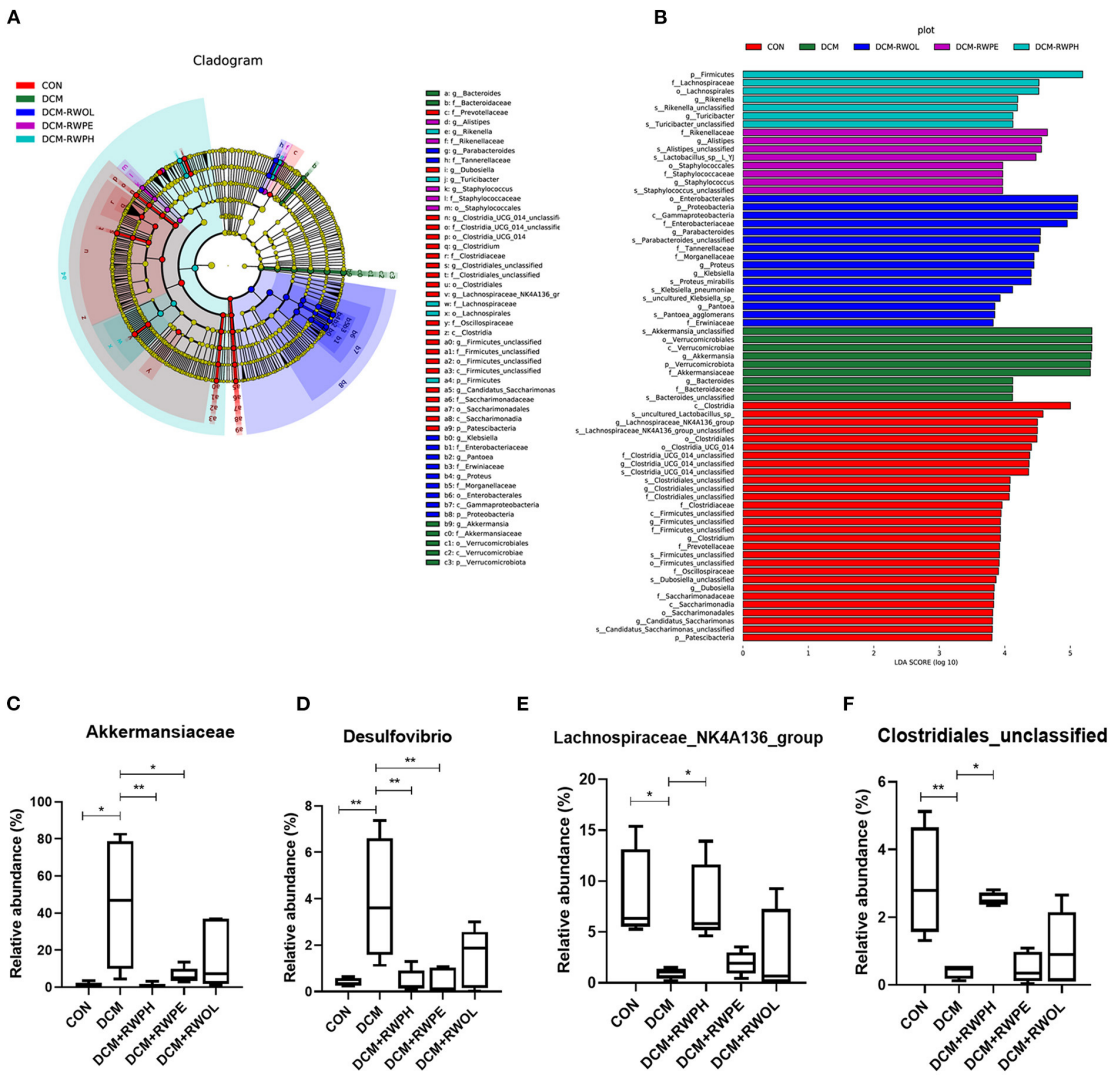
Linear discriminant analysis effect size (LEfSe), linear discriminant analysis (LDA) and abundance comparison of representative gut bacterial taxa. **(A)** Comparison of taxonomic abundances using LEfSe. The circles represent the taxonomic levels from phyla to species. The dots, which size is proportional to its taxonomic abundance, located on individual circles represent different classification levels of bacteria. **(B)** linear discriminant analysis (LDA) represented significant difference in abundance of gut bacteria in the DCM/CON or DCM/DCM + treatment groups. **(C–F)** abundances of representative significantly changed gut taxa among groups. *P < 0.05; **P < 0.01.

We compared the composition of gut microbiota for each group using linear discriminant analysis effect size to identify the specific types of bacterial taxa altered by rice wine functional components. Figure 7A shows the predominant bacteria of each group; the composition of gut microbiota communities differed significantly between the CON group and the DCM group. The composition of gut microbiota communities in the DCM + RWPH group was similar to that of the CON group.

We identified the main bacterial taxa with significant variation between each group by linear discriminant analysis (Figure 7B). At the phylum and genus level, the top three dominant bacteria in the CON group were *Lachnospiraceae*_NK4A136_group, *Clostridia*-UCG-014-unclassified, and *Clostridiales*-unclassified. The abundance of *Verrucomicrobiota* and *Akkermansia* in the DCM group was significantly higher than that of other groups. The top three dominant bacteria in the DCM + RWPH group were *Firmicutes, Rikenella*, and *Turicibacter*. The top three dominant bacteria in the DCM + RWPE group were *Alistipes, Staphylococcus*, and *Staphylococcaceae*. The top three dominant bacteria in the DCM + RWOL group were *Proteobacteria, Enterobacteriaceae*, and *Parabacteroides*.

We selected four taxa that were representative and significantly different among the groups so that we could observe the effect of the functional components of rice wine on disturbances to gut microbiota in the DCM group (Figures 7C–F). The abundance of *Akkermansia* and *Desulfovibrio* was significantly higher in the DCM group than that in the CON group; conversely, the abundance of *Akkermansia* and *Desulfovibrio* dramatically decreased in both the RWPH group and the RWPE group. The abundance of *Clostridiales*-unclassified and *Lachnospiraceae*_NK4A136_group was lowest in the DCM group, but the abundance significantly increased after the intervention of RWPH. The area under the curve for *Akkermansia* and *Desulfovibrio* in the ROC analyses were 0.9375 and 1.0, respectively (Supplementary Figures 1A,B), demonstrating their potential as biological indicators for distinguishing between the CON group and the DCM group.

### RWPH and RWPE modulate the metabolites of gut microbiota in DCM mice

**Figure 8:**
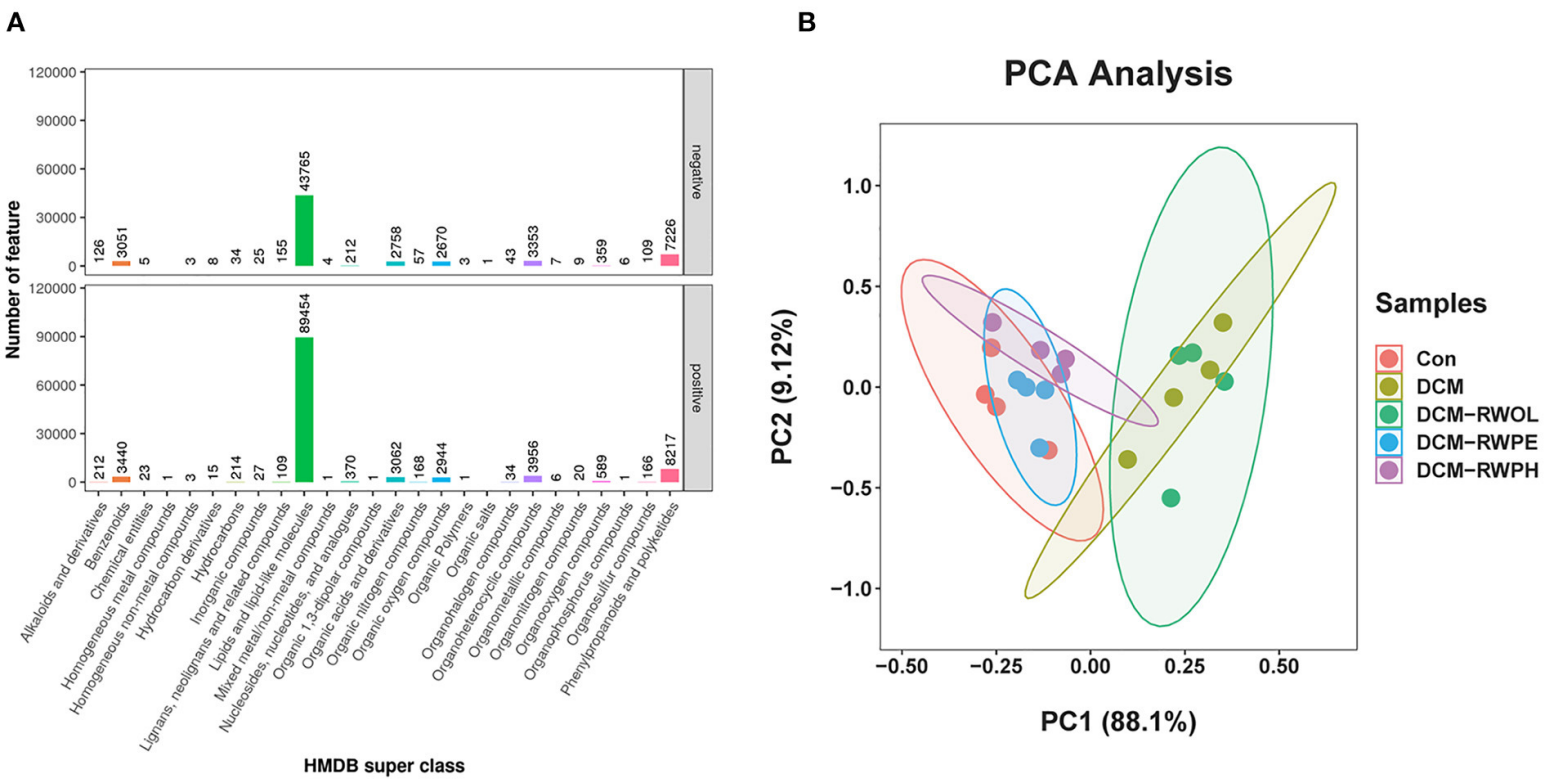
RWPH and RWPE modulated the gut microbiota metabolites in DCM mice. **(A)** Human Metabolome Database (HMDB) super class identification classification and annotation diagram of first-level metabolites. **(B)** Principal component analysis (PCA) analysis of metabolites for 5 groups.

We performed metabolic profiling of serum (*n* = 4, each group) to identify the metabolic phenotypes of gut microbiota that may be associated with each group. Samples were detected by reverse-phase spectrometry in positive and negative ion modes. The results of serum profiling demonstrated 25,521 and 14,186 metabolite features in the positive and negative ion modes, respectively, of which 8,841 and 6,732 were annotated (Supplementary Table 1). According to the Human Metabolome Database super class classification (Figure 8A), lipids and lipid molecules are the most abundant metabolites in both positive and negative ion modes. Principal component analysis was used to observe similarities and differences in the serum metabolites in each group. Principal component analysis results showed significant differences between the CON group and the DCM group (Figure 8B). The serum metabolites of both the DCM + RWPH group and the DCM + RWPE group were similar to those in the CON group, indicating that RWPH and RWPE played a role in alleviating HG-induced metabolic disorders. However, RWOL showed no effect on reversing metabolite disturbances. A volcano plot was used to evaluate the differential metabolites between groups (Supplementary Figures 2A–D). A total of 798 metabolites were upregulated and 495 were downregulated in the DCM group compared to those in the CON group. A total of 483 metabolites were upregulated and 1,021 were downregulated in the DCM group compared to those in the DCM + RWPH group. A total of 456 metabolites were upregulated and 490 were downregulated in the DCM group compared to those in the DCM + RWPE group. A total of 161 metabolites were upregulated and 83 were downregulated in the DCM group compared to those in the DCM + RWOL group.

Bubble charts were created using the KEGG pathway enrichment analysis of the altered metabolites. The RWPE and RWPH treatments significantly affected the biological pathways in the DCM group (Supplementary Figures 3A–D). When we reviewed the Q value, number, and enrichment factors, we identified that the metabolite changes in the DCM group were primarily linked to the choline, tryptophan, and glycerophospholipid metabolism in cancer. Following intervention with RWPH treatment, significantly different metabolites were associated with phenylalanine metabolism, primary bile acids (BAs) biosynthesis, and tryptophan metabolism. After intervention with RWPE treatment, the representative pathways included choline metabolism in cancer, glycerophospholipid metabolism, and linoleic acid metabolism.

**Figure 9:**
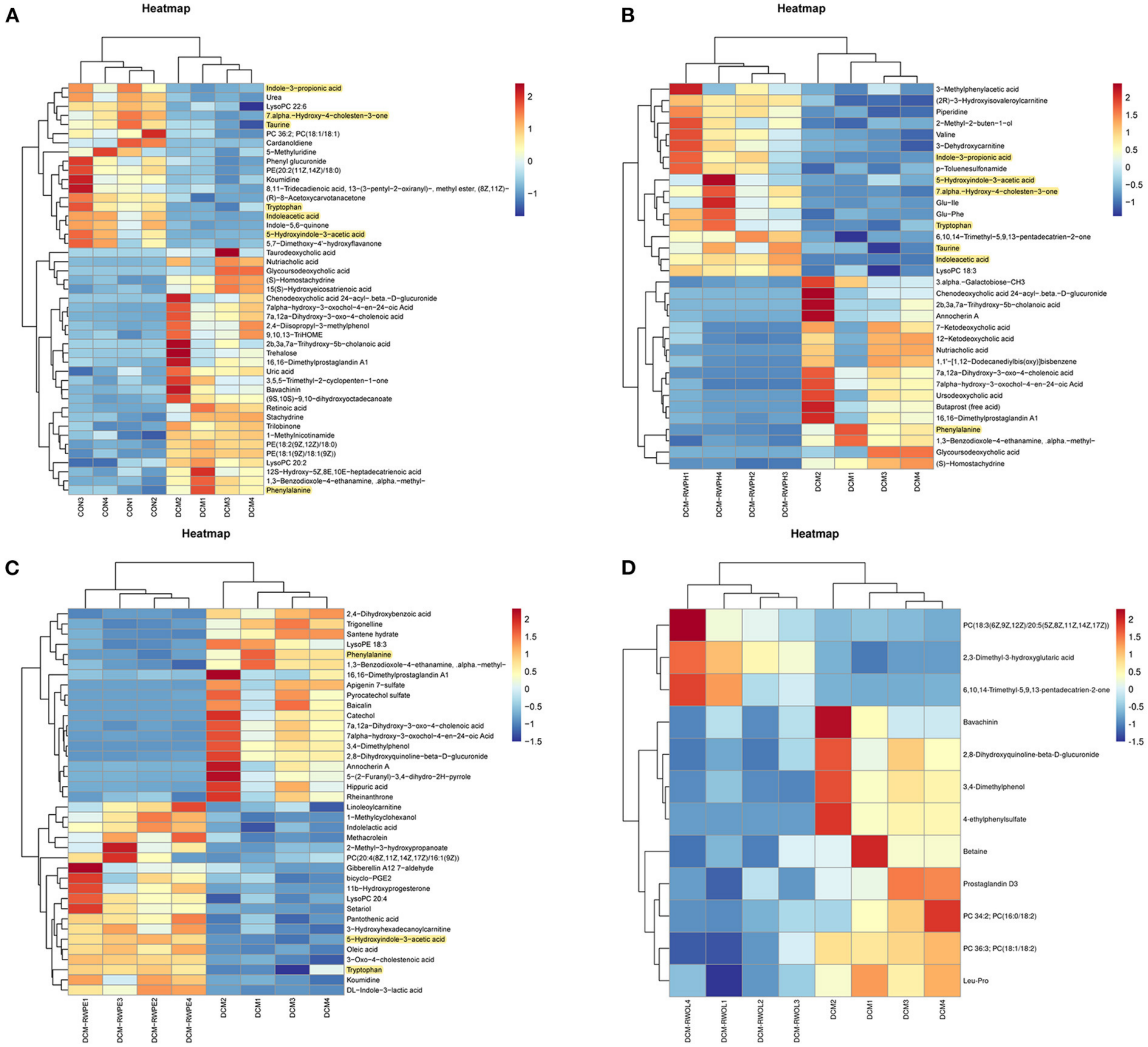
Heat map analysis of significantly different metabolites between groups. **(A–D)** Comparison of significantly altered serum metabolites between the DCM/CON or DCM/DCM + treatment groups by heat map analysis.

**Figure 10:**
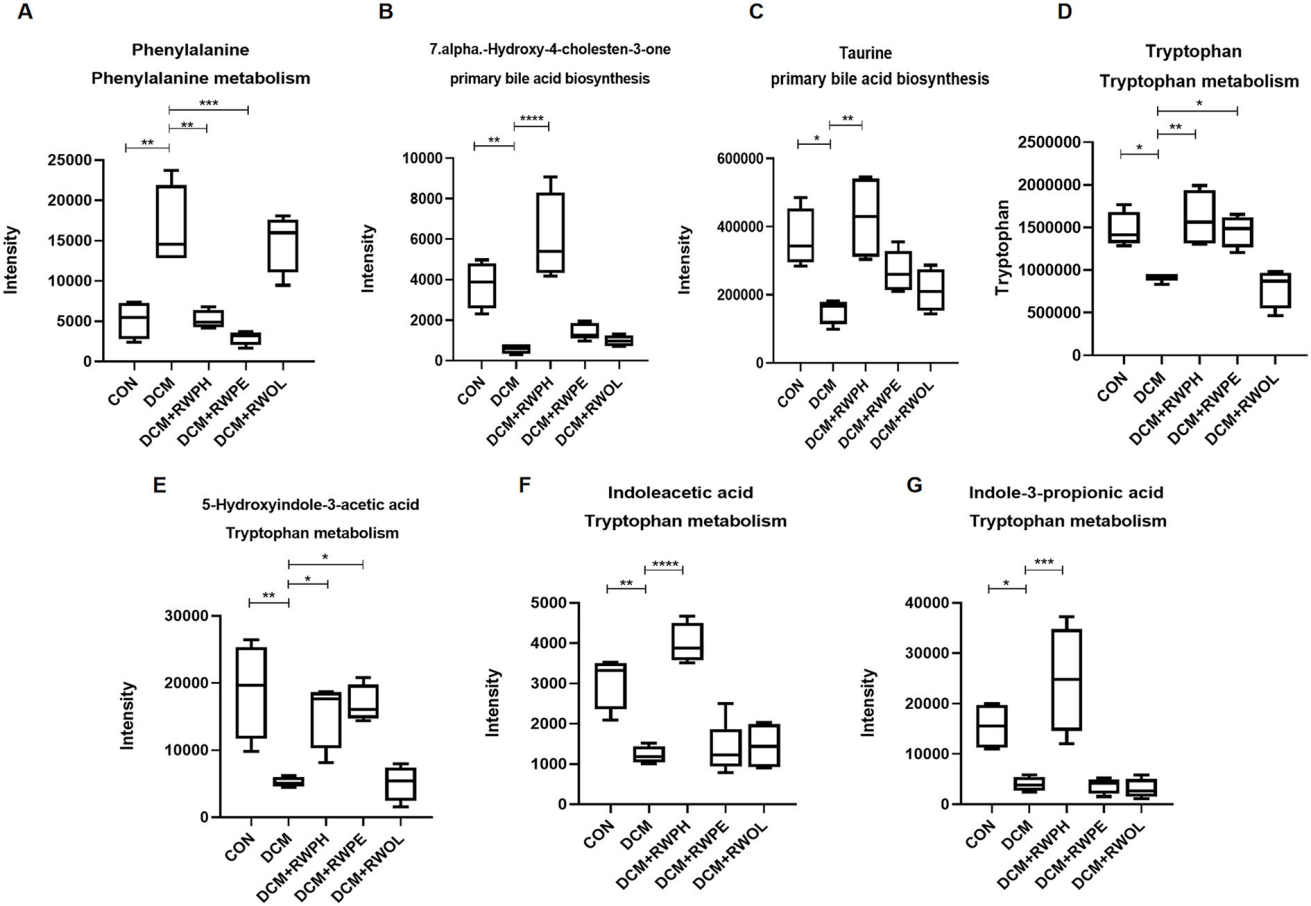
Indensity comparison of representative significantly different metabolites between groups. **(A–G)** Indensity comparison of significantly changed metabolites among groups. *P < 0.05, **P < 0.01, ***P < 0.001, and ****P < 0.0001.

Heatmaps were created to show the differential metabolites between groups (Figures 9A–D). To explore the specific pathways through which RWPH and RWPE play a role in DCM treatment, we combined heatmaps to identify meaningful metabolites with significant differences in the RWPH and RWPE pathways. Phenylalanine was significantly higher in the DCM group compared to that in the CON group, and this effect was reversed after RWPH and RWPE treatment (Figures 10A–G). 7alpha-hydroxy-4-cholesten-3-one, taurine, tryptophan, indoleacetic acid, 5-hydroxyindole-3-acetic acid, and Indole-3-propionic acid were lower in the DCM group, and RWPH and RWPE can play a certain role in increasing these metabolites. Our results indicate that the pathways of primary BAs biosynthesis, phenylalanine metabolism, and tryptophan metabolism could be important to developing new treatments for DCM.

### The connection between the anti-cardiotoxic effects of RWPH and RWPE and gut microbiota and metabolites

We explored the functional correlation between the disturbed gut microbiota and altered circulating metabolites using a correlation analysis based on the Spearman correlation coefficient.

**Figure 11:**
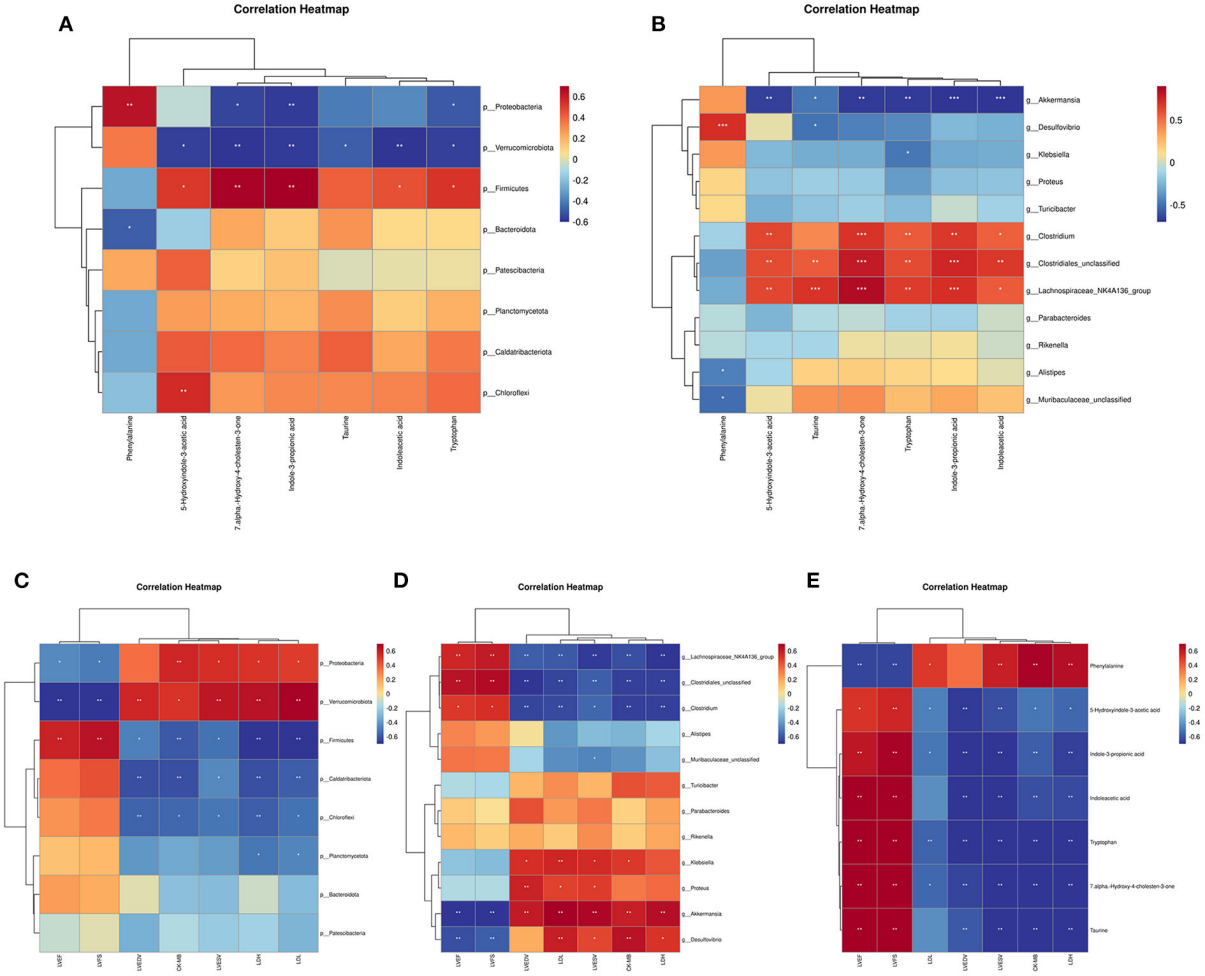
The correlation analysis of gut microbiota, metabolites, cardiac function and myocardial injury parameters. **(A)** Spearman correlation between altered phylum bacterial taxa and altered metabolites. **(B)** Spearman correlation between altered genus bacterial taxa and altered metabolites. **(C)** Spearman correlation between altered bacterial taxa at the phylum level and echocardiographic and myocardial injury parameters. **(D)** Spearman correlation between altered bacterial taxa at the genus level and echocardiographic and myocardial injury parameters. **(E)** Spearman correlation between altered metabolites and echocardiographic and myocardial injury parameters. *P < 0.05, **P < 0.01, and ***P < 0.001.

Results revealed that several gut bacteria were strongly associated with typical metabolites (Figures 11A,B). At the phylum level, *Firmicutes* was positively correlated with 7alpha-hydroxy-4-cholesten-3-one, taurine, tryptophan, indoleacetic acid, 5-hydroxyindole-3-acetic acid, and indole-3-propionic acid. Both *Proteobacteria* and *Verrucomicrobiota* were negatively correlated with 7alpha-hydroxy-4-cholesten-3-one, taurine, tryptophan, indoleacetic acid, 5-hydroxyindole-3-acetic acid, and indole-3-propionic acid. In contrast, phenylalanine was positively correlated with *Proteobacteria* but negatively correlated with *Bacteroidetes*.

At the genus level, *Clostridiales*-unclassified, *Lachnospiraceae*_NK4A136_group, and *Clostridium* were positively correlated with 7alpha-hydroxy-4-cholesten-3-one, taurine, tryptophan, indoleacetic acid, 5-hydroxyindole-3-acetic acid, and Indole-3-propionic acid. *Akkermansia* was negatively correlated with 7alpha-hydroxy-4-cholesten-3-one, taurine, tryptophan, indoleacetic acid, 5-hydroxyindole-3-acetic acid, and Indole-3-propionic acid. In contrast, phenylalanine was positively correlated with *Desulfovibrio* but negatively correlated with *Alistipes* and *Muribaculaceae*-unclassified.

We used a Spearman correlation to establish whether a relationship exists between cardiac function and myocardial injury indicators with intestinal bacteria and metabolites. Results of the Spearman correlation showed an inverse association between certain bacterial taxa (phylum *Proteobacteria*, phylum *Verrucomicrobiota*, genus *Akkermansia*, and genus *Desulfovibrio*) and good cardiac function indices (LVEF and LVFS) (Figures 11C,D). Other bacterial taxa (phylum *Firmicutes*, genus *Clostridiales*-unclassified, genus *Lachnospiraceae*_NK4A136_group, and genus *Clostridium*) showed a positive correlation with good cardiac function indices (LVEF and LVFS) but were negatively correlated with myocardial injury markers and poor cardiac function indicators (Figures 11C,D). A negative correlation was found between good cardiac function markers and phenylalanine levels, whereas a positive correlation was found with the other microbiome-related metabolites that were measured (Figure 11E).

## Discussion

DCM is a pathophysiological condition caused by diabetes mellitus that can lead to heart failure and mortality (24). There are a range of drug therapies that may delay the development of DCM, including GLP-1 agonists, SGLT2 inhibitors, β-blockers and ACEI (25–27); however the efficacy is limited. Polyphenols, polypeptides, and oligosaccharides are present in many foods and beverages, holding antioxidant and anti-inflammatory effects as well as the ability to alter gut microbiota composition (10–13). For example, resveratrol, a red wine polyphenol, exerts cardiovascular protective effects against myocardial fibrosis and atherosclerosis (28, 29). Chinese rice wine is fermented from glutinous rice; it is not only rich in polyphenols but also high in polypeptides and oligosaccharides (6). In this study, in order to explore cardioprotective effects of chinese rice wine functional components, the gut microbiota and microbial-related metabolites were introduced for the treatment of DCM for the first time, with the aim to provide novel insights for DCM therapy.

The functional components in Chinese rice wine have also received extensive attention. Lin et al. found that RWPH prevented doxorubicin-induced cardiotoxicity and improved cardiac function by activating the Nrf2 signaling pathway (9). Meng et al. showed that RWPH and RWPE inhibited vascular smooth muscle cell proliferation and migration (7). In the present study, we found that RWPH and RWPE ameliorated cardiac function in DCM mice as well as alleviated cardiac hypertrophy and myocardial fibrosis at the micro level.

Many published studies have focused on the relationship between DCM and autophagy, given the indication that impaired autophagy has an impact on DCM pathogenesis. As a result of the impaired glucose utilization in DCM, the energy required by the heart is primarily derived from fatty acid oxidation in the mitochondria. The fatty acid oxidation process produces reactive oxygen species, which then causes oxidative stress and mitochondrial dysfunction (30). Autophagy can remove dysfunctional mitochondria that release reactive oxygen species and death-inducing factors, thereby preventing cell damage; an activity that is critical for both cardiac homeostasis and myocardium protection in DCM affected patients (31, 32). In addition to inhibited autophagy, increased cardiac apoptosis is now recognized as a major risk factor for the development of HG-induced cardiomyopathy; heart biopsies from diabetic patients with dilated cardiomyopathy showed an 8-fold higher cardiomyocyte apoptosis than non-diabetic patients who were only affected by dilated cardiomyopathy (33). Apoptosis occurs in the late stage of DCM, eventually leading to impaired cardiac function and remodeling (34). More importantly, there seems to be some crosstalk between autophagy and apoptosis. Recent studies have shown that autophagy can actually protect cells from apoptosis, while activation of the apoptotic pathway can inhibit autophagy (35), suggesting that there is a molecular link between the two, which is a subject for further study.

In our study, we similarly found that DCM is trapped in dysfunction of autophagy and apoptosis. Based on our research results, we suggest that treatment with RWPH and RWPE may reduce the impaired autophagy and excessive apoptosis associated with DCM, thus achieving a certain cardiac protective effect. To further explore the relationship between autophagy and apoptosis, we inhibited the pro-autophagy effect of RWPH and RWPE by 3-MA, and found that reducing autophagy can counteract the anti-apoptotic effect of functional components of rice wine, which also means that RWPH and RWPE may inhibited apoptosis by promoting autophagy. However, we did not explore the relevant molecular mechanism in this study, and we hope to conduct in-depth studies in our subsequent experiments.

Several recent studies have suggested a relationship between cardiovascular diseases and gut microbiota (17). According to the so-called “gut hypothesis of heart failure”, the gut microbiota plays a key role in the progression of chronic heart failure, mainly because of increased intestinal permeability, microbiota translocation (36), significantly reduced microbial composition diversity (37), overgrowth of pathogenic bacteria (36), and unbalanced serum levels of trimethyl-N-oxide (TMAO) and short-chain fatty acids (SCFAs) (38, 39). Heart failure is the most prominent manifestation of DCM, which is a leading cause of mortality (24), indicating that intestinal microecology also plays a crucial role in the development of DCM.

Tsai et al. performed stool 16S-rDNA sequencing and echocardiography in 155 type 2 diabetes patients and found that *Bacteroidetes* and *Firmicutes* at the phylum level were positively correlated with LVEF, and low levels of *Firmicutes* were a risk factor for left ventricular hypertrophy (40). Our results revealed population size reductions and lower α- and β-diversity of the gut microbiota in DCM mice compared to the gut microbiota of CON mice. At the phylum level, the abundance of *Bacteroidetes* and *Firmicutes* decreased in the DCM group compared to that in the CON group, while the abundance of *Verrucomicrobiota* was significantly increased. We observed that treatment with RWPH and RWPE largely prevented DCM-induced gut dysbiosis, especially the reshaping of *Verrucomicrobiota* and *Firmicutes*. Consistent with Tsai's conclusion, we found that the presence of *Firmicutes* was positively correlated with LVEF and LVFS and negatively correlated with myocardial injury markers and poor cardiac function indicators.

We observed elevated populations of *Akkermansia* and *Desulfovibrio* in the gut microbiota of DCM mice, and we found that treatment with RWPH and RWPE significantly reduced the abundance of these microbes. *Akkermansia* is a representative genus of the *Verrucomicrobiota*. There is conflicting evidence regarding the relative abundance of the genus *Akkermansia* in HFD-fed mice and whether *Akkermansia* promotes metabolic disease progression. Xie et al. found that the abundance of *Akkermansia* increased when rats were fed an HF diet (41). *Akkermansia* was more abundant in individuals with type 2 diabetes than in non-diabetic controls (42). Resveratrol reduced the abundance of *Akkermansia* in obese mice (43). Other studies has found that knockout or diet-induced obesity mouse models have reduced the abundance of *Akkermansia* (44). Further research has suggested that *Akkermansia* may maintain the health of the gastrointestinal tract and reduce the risk of diabetes and inflammation (45).

The phylum *Proteobacteria* is elevated in type 2 diabetic patients and HFD-fed mice, suggesting that *Proteobacteria* could be a diagnostic signature of gut dysbiosis and risk of metabolic disease (46). *Desulfovibrio*, which is a genus of the phylum *Proteobacteria*, is known for its sulfate-reducing capacity, thereby producing toxic hydrogen sulfide which can permeate the gut mucus barrier and increase inflammation levels (47).

In this study, *Clostridiales*-unclassified and *Lachnospiraceae*_NK4A136_group were enriched in the CON group and the DCM + RWPH group. Both taxa were strongly positively correlated with LVEF and LVFS and strongly negatively related to negative indicators. Our results support those of Mao et al., who found that *Lachnospiraceae* NK4A136 was significantly decreased in HFD-induced obese mice and was negatively correlated with body weight, blood lipids, and blood glucose (48). Larger populations of *Clostridiales*-unclassified have been described in obesity-resistant rats, and *Clostridiales*-unclassified have been associated with an excellent metabolic phenotype with an associated lower body fat (49). Our results suggest that both RWPH and RWPE play a protective role against DCM by inhibiting the growth of *Akkermansia* and *Desulfovibrio* and that RWPH can promote the growth of beneficial bacteria, such as *Lachnospiraceae*_NK4A136_group and *Clostridiales*-unclassified.

The beneficial effect of RWPH on cardiovascular health may be associated with increased biosynthesis of BAs. In DCM mice, 7alpha-hydroxy-4-cholesten-3-one (an indicator of BA synthesis) increased after treatment with RWPH (50). Primary BAs have been shown to delay disease progression in type 2 diabetic mice by regulating glucose and lipid metabolism and improving insulin sensitivity (51). Moreover, the body uses cholesterol to synthesize new primary BAs, suggesting that activation of the primary BAs biosynthesis pathway represents further depletion of circulating cholesterol. Taurine is the raw material of secondary BAs and the presence of taurine is known to regulate cholesterol (52). The increase in taurine levels after RWPH intervention, suggests that RWPH treatment can reduce the levels of circulating cholesterol by promoting primary BAs synthesis. This delivers a cardiovascular protective effect and is evidenced by the low level of low-density lipoprotein cholesterol that we observed in the DCM + RWPE group.

Tryptophan is an essential amino acid in humans, and tryptophan metabolism is regulated by gut microbiota. The metabolites of tryptophan have immunological, metabolic, and neuroregulatory functions, which are current therapeutic targets for various diseases (53). Tryptophan metabolism is mainly composed of three pathways: kynurenine, serotonin, and the indole/AhR pathway (54). In our experiments, the intermediate or final products of tryptophan metabolic pathways were significantly decreased in the DCM group, however the levels increased after RWPH and RWPE treatments. This suggests that RWPH and RWPH can promote tryptophan metabolism. In addition, we found that phenylalanine (existing the phenylalanine metabolism pathway) was increased in the DCM group, and the levels decreased after RWPH and RWPE treatments. An increasing number of studies have confirmed that phenylalanine is associated with the risk of diabetes and cardiovascular diseases (55, 56), indicating that phenylalanine metabolism may be a breakthrough to improving DCM outcomes.

Our study had fundamental limitations. Firstly, the number of experimental mice in each group was not of a sufficient sample size, and there may be individual differences between mice that cannot be accounted for in the current study. On repetition, the data and results of this study may deviate with a larger sample size. Secondly, although our experiments demonstrated that RWPH and RWPE may inhibit apoptosis by promoting autophagy, thereby exerting cardioprotective effects, we did not study the relevant mechanisms in depth, nor did we correlate the results of autophagy and apoptosis with gut microbiota and its metabolites. In future experiments, we will investigate the relationships that may exist between DCM and autophagy, apoptosis, and gut microbiota. Thirdly, we did not investigate whether the improvement of DCM by treatment with RWPH and RWPE is dependent only on the changes in gut microbes and related metabolites. In the following experiments, we will determine whether the depletion of gut microbes negates the cardioprotective effects of RWPH and RWPE by adding a treatment group that is depleted of gut. Finally, a literature review has indicated that transplantation of fecal microbiota is the most effective way to rebalance gut microbiota (57). In subsequent work, we will investigate the potential role of fecal microbiota transplantation to improving DCM outcomes.

## Conclusions

Our study used 16S-rDNA sequencing, non-targeted metabolomic profiling, and markers of cardiac function and myocardial injury to investigate the relationship between DCM and the functional components of Chinese rice wine. We found that RWPH and RWPE may have a cardioprotective effect against DCM by modulating the composition and metabolic function of the gut microbiota. Specifically, RWPH increased the abundance of the gut microbes *Clostridiales*-unclassified and *Lachnospiraceae*_NK4A136_group and decreased the abundance of *Akkermansia* and *Desulfovibrio*. Our results showed that RWPH increased the biosynthesis of primary BAs and tryptophan metabolism-associated metabolites and reduced serum phenylalanine. RWPE reduced the abundance of *Akkermansia* and *Desulfovibrio*. Similar to RWPH, RWPE increased tryptophan metabolism-associated metabolites and reduced serum phenylalanine. Both RWPH and RWPE improved the cardiac function of mice with DCM by promoting protective autophagy and inhibiting cardiomyocyte apoptosis. This article indicates potential novel therapeutic options for DCM by use of foods that enhance the health of gut microbiota and related metabolites.

## Data availability statement

The original contributions presented in the study are included in the article/Supplementary materials, further inquiries can be directed to the corresponding author.

## Ethics statement

The animal study was reviewed and approved by Laboratory Animal Ethics Committee of the First Affiliated Hospital of Shaoxing University.

## Author contributions

JY, JS, and JC designed and drafted the manuscript. HG and JC provided essential reagents and materials. JY, JS, JZ, and HL conducted laboratory assays. ZW, NL, WX, and JC analyzed and integrated the data. All authors read and approved the final manuscript.

## Funding

This work was supported by grants from the National Natural Science Foundation of China (Nos. 82000252 and 81873120), the science and technology plan special project of Shaoxing city (No. 2020B33002), and the program for application of public welfare technology of Shaoxing city (No. 2020A13015).

## Conflict of interest

The authors declare that the research was conducted in the absence of any commercial or financial relationships that could be construed as a potential conflict of interest.

## Publisher's note

All claims expressed in this article are solely those of the authors and do not necessarily represent those of their affiliated organizations, or those of the publisher, the editors and the reviewers. Any product that may be evaluated in this article, or claim that may be made by its manufacturer, is not guaranteed or endorsed by the publisher.

## Abbreviations

DCM: diabetic cardiomyopathy
RWPH: rice wine polyphenols
RWPE: rice wine polypeptides
RWOL: rice wine oligosaccharides
LVEF: left ventricular ejection fraction
LVFS: left ventricular fractional shortening
LVEDV: left ventricular end-diastolic volume
LVESV: left ventricular end-systolic volume
CK-MB: creatine kinase-MB
LDH: lactate dehydrogenase
LDL-C: low-density lipoprotein cholesterol
PCoA: principal coordinates analysis
LEfSe: linear discriminant analysis effect size
LDA: linear discriminant analysis
HMDB: Human Metabolome Database
PCA: principal component analysis
BAs: primary bile acids
TMAO: trimethyl-N-oxide
SCFAs: short-chain fatty acids
3-MA: 3-Methyladenine.
