# Supplementary material for: Functional components of Chinese rice wine can ameliorate diabetic cardiomyopathy through the modulation of autophagy, apoptosis, gut microbiota, and metabolites
Source: Front Cardiovasc Med. 2022 Sep 14;9:940663. doi: 10.3389/fcvm.2022.940663 (PMC9515449; doi:10.3389/fcvm.2022.940663)
Supplement: Supplementary file 1 [file Data_Sheet_1.PDF]

Supplementary Methods

DNA extractions and 16S rDNA sequencing

DNA from different samples was extracted using the E.Z.N.A. Stool DNA Kit(D4015, Omega, Inc., Norcross, GA, USA) in accordance with the manufacturer's instructions. Total DNA was eluted in 50μL of elution buffer and stored at -80 °C until measurement by LC-Bio Technology Co., Ltd (Hangzhou,Zhejiang Province, China). The primers used were 341F(5'-CCTACGGGNGGCWGCAG-3') and 805R(5'-GACTACHVGGGTATCTAATCC-3'). The 5' ends of the primers were tagged with specific barcods per sample and sequencing universal primers. PCR amplification was performed in a total volume of 25 uL reaction mixture containing 25 ng of template DNA，12.5 uL PCR Premix,2.5 uL of each primer,and PCR-grade water to adjust the volume. The PCR conditions to amplify the prokaryotic 16s fragments consisted of an initial denaturation at 98 °C for 30 seconds; 32 cycles of denaturation at 98 °C for 10 seconds, annealing at 54°C for 30 seconds, and extension at 72°C for 45 seconds; and then final extension at 72°C for 10 minutes. The PCR products were confirmed with 2% agarose gel electrophoresis. Throughout the DNA extraction process, ultrapure water, instead of a sample solution, was used to exclude the possibility of false-positive PCR results as a negative control. The PCR products were purifyied by AMPure XT beads(Beckman Coulter Genomics, Danvers, MA, USA) and quantified by Qubit (Invitrogen,USA). The amplicon pools were prepared for sequencing and the size and quantity of the amplicon library were assessed on Agilent 2100 Bioanalyzer (Agilent, uSA) and with the Library Quantification Kit for lllumina (Kapa Biosciences, Woburn, MA, USA), respectively. The libraries were sequenced on NovaSeq PE250 platform.Samples were sequenced on an Illumina NovaSeq platform (Illumina, San Diego, CA, USA) according to the manufacturer's recommendations.

Untargeted metabolomics analysis

The collected serum samples were thawed on ice, and metabolites were extracted with 50% methanol buffer. Briefly, 20μL of the sample was extracted using 120μL of precooled 50% methanol, vortexed for 1 min, and incubated at room temperature for 10 min. The extraction mixture was then stored overnight at -20 °C. After centrifugation at 4,000 × g for 20 min, the supernatants were transferred into new 96-well plates and stored at -80 °C prior to LC-mass

spectrometry (MS) analysis. Pooled quality control samples were prepared by combining 10 μL of each extraction mixture[21]. All samples were acquired using the LC-MS system following machine orders. A high-resolution tandem mass spectrometer TripleTOF5600plus (SCIEX, Mereside, UK) was used to detect metabolites eluted from the column. Quadrupole time-of-flight MS was operated in both positive and negative ion modes. Pretreatments of the acquired MS data, including peak picking, peak grouping, retention time correction, second peak grouping, and annotation of isotopes and adducts, were performed using the XCMS software. CAMERA and metaX toolbox were implemented using the R software. Each ion was identified by combining the retention time and m/z data. Peak intensities were recorded, and a three-dimensional matrix containing arbitrarily assigned peak indices, sample names, and ion intensity information was generated. The online Kyoto Encyclopedia of Genes and Genomes (KEGG) database was used to annotate the metabolites by matching the exact molecular mass data (m/z) of samples with those from the database.

Supplemental Figures and Figure Legends

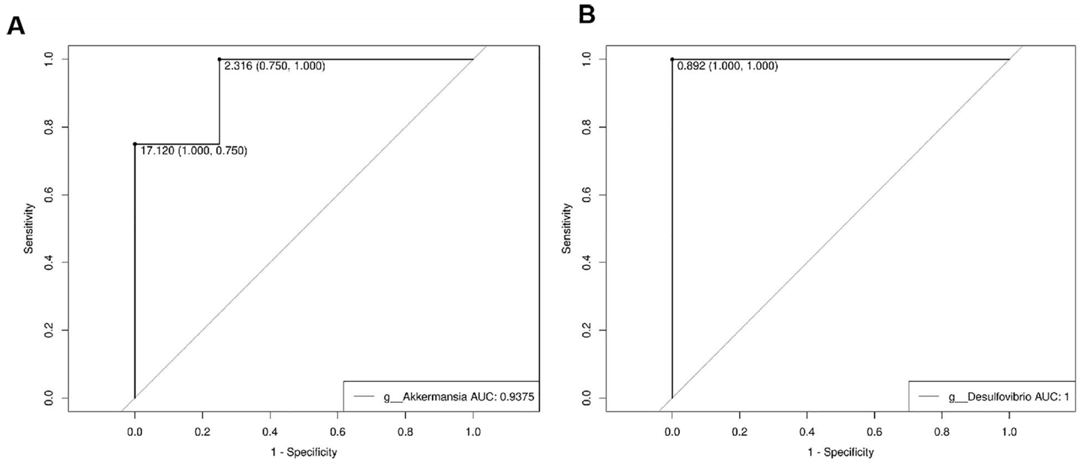

Figure 1 A-B, ROC curve showed diagnostic role of Akkermansia and Desulfovibrio distinguishing the DCM and the Con group. The area under the curve (AUC) values of Akkermansia and Desulfovibrio were 0.9375 and 1, respectively.

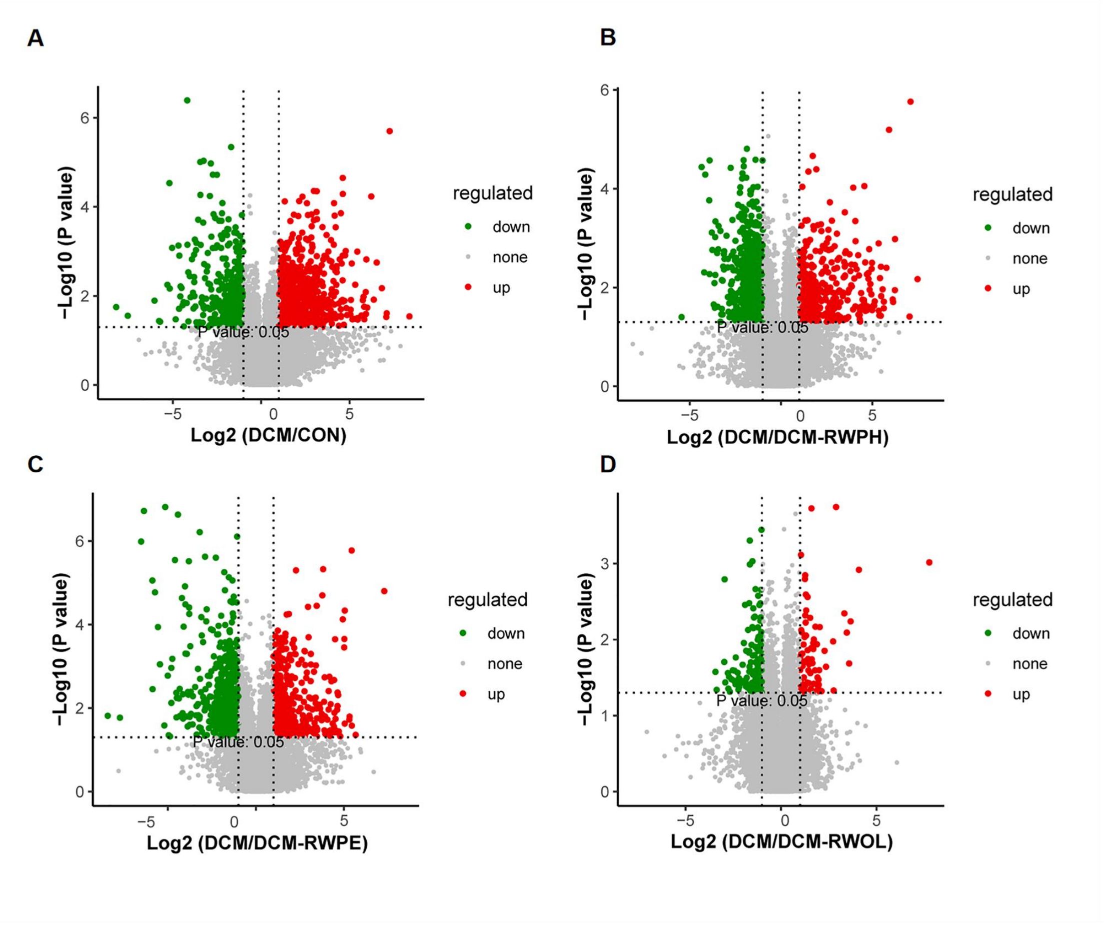

Figure 2 A-D, Volcano plots of the differential metabolites in the DCM/CON or the DCM/DCM+treatment groups.

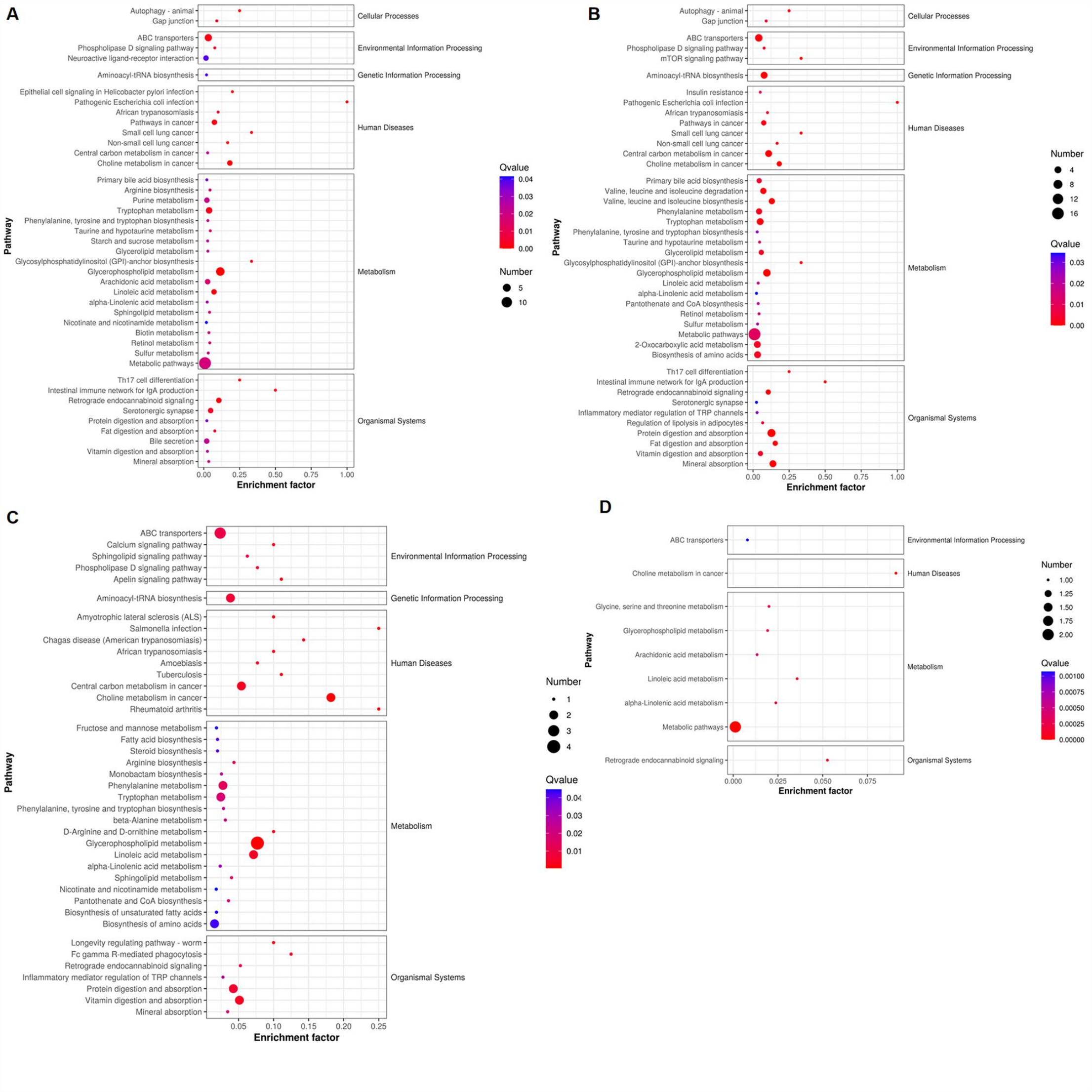

Figure 3: DCM and treatments of RWPE and RWPH significantly affect the biological pathways. A-D, KEGG enrichment pathway analysis of significant different metabolites in the DCM/CON or the DCM/DCM+treatment groups.

Supplemental Table

Table 1

Identification statistics					
mode	All feature	Annotated	MS2	HMDB	KEGG
negative	14186	6732	552	5624	4523
positive	25521	8841	612	7476	5368
